# Effects of the tobacco–maize relay intercropping pattern on soil nutrients and soil microbial diversity

**DOI:** 10.3389/fmicb.2024.1389156

**Published:** 2025-01-10

**Authors:** Kang Yang, Shuhui Zi, Chengren Ouyang

**Affiliations:** College of Tobacco Science, Yunnan Agricultural University, Kunming, Yunnan, China

**Keywords:** relay intercropping, soil nutrients, soil microbial diversity, tobacco, maize

## Abstract

The imbalanced soil nutrient status caused by the long-term monoculture of flue-cured tobacco are a concern. The tobacco–maize relay intercropping, widely used in Yunnan, may improve soil nutrients by enhancing the soil microbial community, but this remains unexplored. This study employed high-throughput sequencing technology to examine soil microbial diversity under tobacco monoculture and tobacco–maize relay intercropping, using the varieties Hongda and K326, respectively. The results indicated that tobacco–maize relay intercropping significantly enhanced root biomass compared to tobacco monoculture, with no significant effect on aboveground biomass. This intercropping treatment also significantly improved soil physicochemical properties, including soil pH, total phosphorus, available phosphorus, and available potassium, which was associated with an increase in the soil microbial community (as indicated by the Chao1 and Shannon indices). Specifically, the abundance of arbuscular mycorrhizal fungi, Nitrospira, and Acidobacteria increased, but the abundance of Chloroflexi decreased. Therefore, these findings suggest that tobacco-maize relay intercropping can improve soil physicochemical properties and enhance soil nutrient supply.

## Highlights

• Tobacco–maize relay intercropping improved soil physicochemical properties.
• Tobacco–maize relay intercropping can improve the soil microbial community richness and diversity (Chao1 index and Shannon index).
• Redundancy analysis suggested that the soil physicochemical properties significantly affected the soil community composition.

## 1 Introduction

Tobacco, a significant cash crop globally, is extensively cultivated in both developed and developing countries (Ye et al., 2022). In China, intensive agricultural practices have been implemented to optimize water and fertilizer management in tobacco fields (Smith and Siciliano, 2015). However, these practices have led to a series of soil-related issues over recent decades, including soil fertility degradation and biodiversity loss (Lai et al., 2017). Compounding these problems, the prevalence of soil-borne tobacco diseases has escalated due to prolonged monoculture practices (She et al., 2017), resulting in decreased tobacco yields and reduced economic returns for farmers. Most studies indicate a positive correlation between soil microorganism diversity and soil fertility (Gao et al., 2014; Ferran et al., 2023). Therefore, enhancing the diversity of soil microorganism communities is a crucial strategy to improve soil fertility and boost tobacco yields.

Relay intercropping, a significant pattern utilized globally to enhance crop yields, demonstrates improved soil resource utilization efficiency (Wu et al., 2022). A novel and resource-use efficient pattern of maize intercropped with soybean strips has been suggested that this pattern significantly boosted land-use efficiency and conserves fertilizer (Tan et al., 2021; Gao et al., 2014; Li et al., 2019). This system offers increased income for farmers compared to monoculture planting. The pattern was widely recognized in adjusting the agricultural industry structure and promoting sustainable agriculture development (Zhou et al., 2019; Misra et al., 2019). In relay intercropping patterns, the primary challenge involves competition and sharing of soil nutrient resources, which impact root nutrient absorption, subsoil nutrient utilization, and overall crop growth and development (Liu et al., 2021; Li et al., 2019). Numerous studies have demonstrated that relay intercropping effectively mitigates soil nutrient decline and imbalance (Tan et al., 2021; Liu et al., 2021), ameliorates soil physicochemical properties, and bolsters soil enzyme activity relative to prolonged monoculture (Mayberry et al., 2017; Workayehu and Wortmann, 2011). For example, Xu et al.’s (2021) result showed that maize intercropped with alfalfa improved the soil physicochemical properties, facilitated nitrogen absorption and increased crop yield. Moreover, relay intercropping patterns have proven advantageous in reducing ammonia volatilization and N_2_O emissions, increasing nitrate nitrogen accumulation in the topsoil, and supplying adequate nitrogen sources for crop nitrogen uptake (Chen et al., 2019). This implies that such a pattern enhances land productivity as soil nutrients increased. Nevertheless, the mechanisms by which relay intercropping influences soil nutrients remain elusive.

Soil microorganisms, integral to agroecosystem functioning, significantly contribute to soil nutrient supply during their formation, recycling, and fertility development processes (Garbeva et al., 2004; Jangid et al., 2008). This contribution is partly due to the enhanced soil structure and fertility resulting from increased soil microorganism diversity (Luan et al., 2021). Such diversity serves as a comprehensive indicator of soil health or nutrient fertility (Li et al., 2018). Previous studies predominantly examined the relationship between soil microbial diversity and nutrients, encompassing the abundance, composition, and structure of soil microbial communities (Zhang et al., 2018; Cui et al., 2021). Numerous factors, including substrate quantity/quality, soil pH, types, and texture, have been identified to influence soil microbial communities (Garbeva et al., 2004; Jangid et al., 2008). Furthermore, climate change, agricultural management, and plant diversity and community composition are closely linked to soil microbial community diversity (Schmid et al., 2021). However, there is limited research on soil microorganisms under flue-cured tobacco relay intercropping systems. It is unclear whether relay intercropping patterns can enhance soil microbial diversity and improve soil ecological function. The following hypothesis was tested in this study: Relay intercropping can alter soil physicochemical properties by enriching the soil bacterial and fungal community composition. The objective was to investigate the characteristics of soil nutrients and their effects on soil microbial diversity in flue-cured tobacco and maize intercropping systems, thereby providing a reasonable practical significance for tobacco field layout and nutrient management.

## 2 Material and methods

### 2.1 Experimental sites

The teaching farm of Yunnan Agricultural University (N 25°18′, E 102°45′, 1930 m.a.s.l) served as the experimental site. This region features a low-latitude plateau monsoon climate, with an average temperature of 14.5°C and rainfall of 960 mm annually, primarily between May and October, and 2,617 h of sunshine per year. The site’s red soil (Chinese classification standard) has a silty clay loam texture, composed of 10% sand (2,000–60 μm), 40% silt (60–2 μm), and 50% clay (< 2 μm). The soil’s physical and chemical properties include: total nitrogen, 0.19 g⋅kg^–1^; total phosphorus, 0.12 g⋅kg^–1^; total potassium, 0.18 g⋅kg^–1^; available phosphorus, 24.13 mg⋅kg^–1^; available potassium, 86.35 mg⋅kg^–1^; available nitrogen, 81.29 mg⋅kg^–1^; and a pH of 6.66.

### 2.2 Experimental design

**FIGURE 1:**
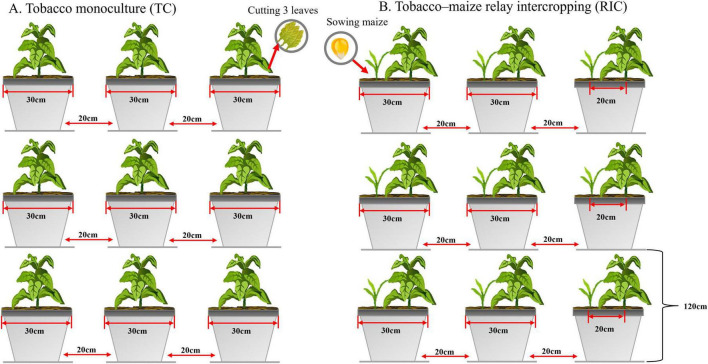
Design of the field experiment at the study site. A total of three treatments were applied. TC, Tobacco monoculture, RIC, Tobacco–maize relay intercropping.

The experiment employed a double-factor design with four treatments: Hongda monoculture (HM), Hongda relay-intercropped maize (HR), K326 monoculture (KM), and K326 relay-intercropped maize (KR). Each treatment was repeated ten times in the pot experiment, following the field layout (Figure 1).

The flue-cured tobacco varieties, K326 and Hongda, and the maize variety, Dianyu 888, were utilized. From March to April 2021, the tobacco company germinated the flue-cured tobacco seedlings, which were subsequently transplanted on May 9, 2021. During transplanting, each plant received 10 g of compound fertilizer (N-P_2_O_5_-K_2_O = 12-6-24), with additional applications of 5 and 10 g per plant at 15 and 37 d post-transplanting, respectively. Weeds were removed manually at 15, 27, 39, and 55 d post-transplanting. Upon leaf maturation, and with 18–20 effective leaves remaining after plant topping, two to three lower leaves were harvested for curing. Maize seeds were sown on August 15, 2021, without initial fertilization. Instead, the maize seedlings received 10 g and 15 g of urea per plant at 15 and 45 days post-sowing, respectively. Weeds were removed manually at 30 and 49 days after maize seedling emergence.

### 2.3 Plant and soil sampling

#### 2.3.1 Plant sampling

At the maturity stage (80 d post-transplanting), three tobacco plants were marked and selected in 2021. Biomass samples from these plants were initially placed in a constant-temperature oven at 105°C for 2 h, then the temperature was reduced to 85°C until a constant weight was achieved. Subsequently, the dried and cooled samples were weighed using an electronic balance, pulverized through a 40-mm sieve, and stored in sealed bags. These prepared samples were then utilized for the analysis of plant nutrient contents.

#### 2.3.2 Soil sampling

At the maturity stage of tobacco in 2021, soil samples were collected, with the loose soil around the roots removed and the remaining soil thoroughly mixed. From each plot, five samples were randomly selected and combined to create a homogenous composite sample, a process repeated for all three replications in each planting mode. These samples were promptly preserved in a portable storage box and transported to the lab. A segment of each soil sample was air-dried, with visible gravel, plant roots, and debris removed, before being ground and sieved through a 2.0-mm mesh for basic nutrient analysis. Another segment was stored at −80°C for soil microbial DNA extraction, while the final segment was maintained at 4°C to assess the soil’s physicochemical properties.

### 2.4 Indexes and method of measurement

#### 2.4.1 Determination of tobacco biomass and plant nutrients

Upon reaching maturity, three tobacco plants were randomly selected, with their roots, stems, and leaves weighed separately in their fresh state. Subsequently, the samples underwent a 30-min oven treatment at 105°C, followed by drying at 85°C to obtain their dry weight. The crushed plant samples were then analyzed for their nitrogen, phosphorus, and potassium contents. The total nitrogen was determined using the Kjeldahl method, while total phosphorus was assessed using the vanadium-molybdenum yellow colorimetric method (Ruan et al., 2021; Zhao et al., 2022). Finally, flame atomic absorption spectrophotometry was used to quantify total potassium (Ahmet, 2013).

#### 2.4.2 Soil nutrient content determination

The soil pH was determined using the potentiometric method (IQ150 pH meter, SPECTRUM, USA). Effective phosphorus content was assessed via a molybdenum antimony anti-spectrophotometer, while fast-acting potassium content was determined using an atomic absorption flame spectrophotometer. Fast-acting nitrogen content was evaluated through alkaline distillation, and total nitrogen content was ascertained using the concentrated sulfuric acid digestion method. The molybdenum blue colorimetric method was employed to quantify total phosphorus, and a flame photometer was used for total potassium content determination. Furthermore, soil peroxidase activity was measured using potassium permanganate titration; sucrase activity, via the 3,5-dinitro salicylic acid colorimetric method (Safarik and Prochazkova, 2022); urease activity, using the sodium phenol-sodium hypochlorite colorimetric method (Zhong et al., 2024); and neutral phosphatase activity, through the sodium benzene phosphate colorimetric method (Mohamed et al., 2021).

#### 2.4.3 DNA extraction, polymerase chain reaction (PCR) amplification, and Illumina Hiseq 2500 sequencing

DNA extraction and PCR amplification total DNA was extracted from 0.5 g of each soil sample using a Fast DNA Spin Kit for soil by MO BIO Biomedicals (USA). The quantity of the extracted DNA was determined using a Nano Drop 2000 Spectrophotometer. Polymerase chain reaction (PCR) was performed using specific primer sets for the bacterial (27F and 1492R) and fungi (ITS1F and ITS2). The DNA was tested for quantity and quality with a spectrophotometer (NanoDrop ND 2000, USA) and by 2% agarose gel electrophoresis, respectively. The resulting amplicon was sequenced using the Illumina MiSeq platform and generated approximately 250 bp paired-end reads. The raw sequencing data was analyzed using the QIIME2 software (Eswaran and Khandeparker, 2017; Toju et al., 2012).

The PCRs were carried out in reaction mixtures containing 10 ng of soil bacterial/fungal DNA, 2 mL of 2.5 mmol/L dNTPs, 0.8 ml of each primer (5 mmol/L), 0.4 ml Fast-Pfu Polymerase and supplemented with 20 ml dd H_2_O. Reactions were run for 35 cycles at 95°C for 30 s, 58°C for 30 s, and 72°C for 1 min, followed by a final extension step at 72°C for 5 min and held at 4°C. PCR products were analyzed by gel electrophoresis in a 2% agarose gel.

### 2.5 Statistical analysis

The optimized sequences, obtained post-splicing, quality control, and de-splicing, were subjected to operational taxonomic unit (OTU) clustering with 97% similarity. This process yielded OTU abundance tables for subsequent confidence analysis. The richness index Chao and diversity index Shannon was calculated using the “vegan” package in R for performing Alpha diversity analysis, as along with bacterial community distribution, clustering analysis, and bacterial function prediction analysis.

## 3 Results

### 3.1 Characteristics of tobacco biomass under tobacco–maize relay intercropping

**FIGURE 2:**
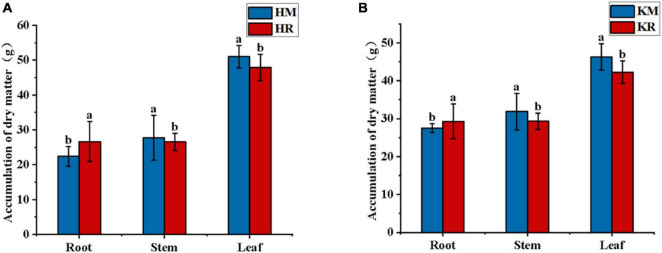
The biomass of tobacco in the relay tobacco-maize intercropping system. Values represent the mean. Different lower-case letters indicate significant differences at *P* < 0.05 (LSD). **(A,B)** represent Honda, K326, respectively.

Figure 2 illustrates the impact of tobacco–maize relay intercropping on tobacco biomass. Compared with Hongda monoculture, Hongda intercropped with maize exhibited a 19.05% increase in root biomass (*P* < 0.05), whereas stem and leaf biomass decreased by 4.32 and 6.12%, respectively (*P* < 0.05). Similarly, K326 intercropped with maize showed a 6.55% increase in root biomass (*P* < 0.05, stem and leaf biomass decreasing by 8.05 and 8.71%, respectively). Both varieties demonstrated increased root weight under tobacco–maize relay intercropping, alongside decreased stem and leaf weight. This suggests that tobacco–maize relay intercropping patterns enhances tobacco root development, thereby augmenting the root biomass of flue-cured tobacco.

### 3.2 Characteristics of soil physicochemical properties under tobacco–maize relay intercropping

Table 1 presents the physicochemical properties of soil under tobacco–maize relay intercropping. Compared with Hongda monoculture, Hongda tobacco intercropped with maize exhibited significant increases in soil pH, total phosphorus, available potassium, available phosphorus, and available nitrogen by 11.87, 16.72, 36.86, 7.11, and 4.87%, respectively (*P* < 0.05). Conversely, the total potassium and total nitrogen contents decreased by 14.79 and 3.78%, respectively (*P* < 0.05). K326 intercropped with maize also showed significant increases in soil pH, total phosphorus, available phosphorus, and available nitrogen by 12.76, 10.72, 12.09, and 0.997%, respectively (*P* < 0.05), compared with K326 monoculture, while potassium, total nitrogen, and available potassium contents decreased by 3.42, 6.64, and 15.34%, respectively (*P* < 0.05). Both tobacco varieties intercropped with maize demonstrated increases in soil pH, total phosphorus, available phosphorus, and available nitrogen, indicating that tobacco–maize relay intercropping enhances soil acid–base environment, nutrient capacity, nutrient absorption by tobacco plants, and nutrient usage efficiency.

**TABLE 1:** Comparison of soil physical and chemical properties under different treatments.

Species	Treatment	Soil pH	Total *N* (TN, g kg^–1^)	Total *P* (TP, g kg^–1^)	Total *K* (TK, g kg^–1^)	Available *P* (AP, mg kg^–1^)	Available *K* (AK, mg kg^–1^)	Available *N* (AN, mg kg^–1^)
HD	HM	6.04 ± 0.02b	1.32 ± 0.05a	2.11 ± 0.63b	4.26 ± 0.51a	74.57 ± 2.06b	143.46 ± 12.50b	139.81 ± 3.52b
	HR	6.75 ± 0.04a	1.27 ± 0.08b	2.47 ± 0.03a	3.63 ± 0.15a	79.87 ± 2.44a	196.33 ± 34.03a	146.61 ± 5.99a
K326	KM	6.03 ± 0.03b	1.40 ± 0.11a	2.20 ± 0.05b	3.70 ± 0.35a	74.70 ± 3.65b	271.38 ± 60.41a	155.95 ± 8.84a
	KR	6.80 ± 0.19a	1.31 ± 0.03a	2.44 ± 0.47a	3.57 ± 0.43a	83.73 ± 3.65a	229.75 ± 27.08b	157.50 ± 13.46a

Values represent means ± standard errors. Different lower-case letters indicate significant differences at *P* < 0.05 (LSD).

### 3.3 Characteristics of the soil enzyme activity of planted tobacco soils under tobacco–maize relay intercropping

**FIGURE 3:**
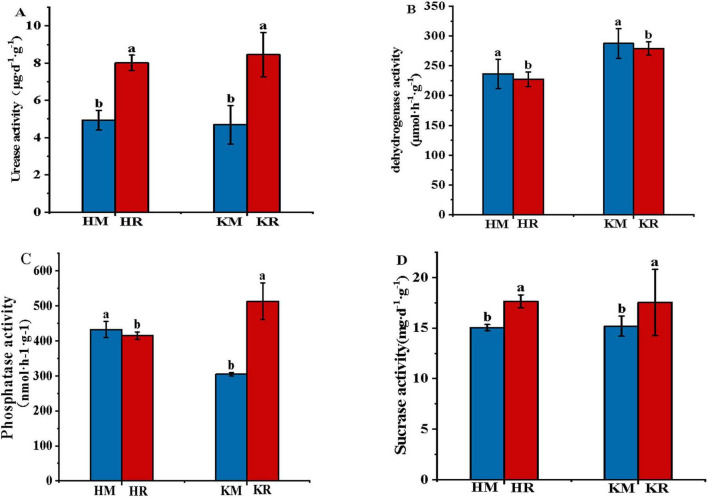
The soil enzyme activities in the relay tobacco-maize intercropping (*n* = 10). Values were mean ± standard deviation, different letters behind the valve indicated a factor, and the difference was statistically significant (*P* < 0.05). **(A,B,C,D)** represent soil urease activity, soil catalase activity, soil catalase phosphatase activity, soil sucrase activity, respectively.

Figure 3 showed the soil enzyme activity of tobacco–maize relay intercropping. Compared with the Hongda monoculture, the Hongda–maize relay intercropping pattern significantly enhanced soil urease activity by 62.37% and soil invertase activity by 17.34%, while reducing soil phosphatase and catalase activities by 3.84 and 4.08%, respectively (*P* < 0.05). Similarly, the K326 relay intercropping pattern increased soil urease, invertase, and phosphatase activities by 80.24, 15.41, and 68.43%, respectively, and decreased soil catalase activity by 2.95%, relative to the K326 monoculture (*P* < 0.05). These results suggested that tobacco–maize relay intercropping patterns promoted soil urease and invertase activities and reduced the soil catalase, thereby increasing soil nutrient supply and facilitating nutrient absorption of plants.

### 3.4 Characteristics of soil microbial communities under tobacco–maize relay intercropping

**FIGURE 4:**
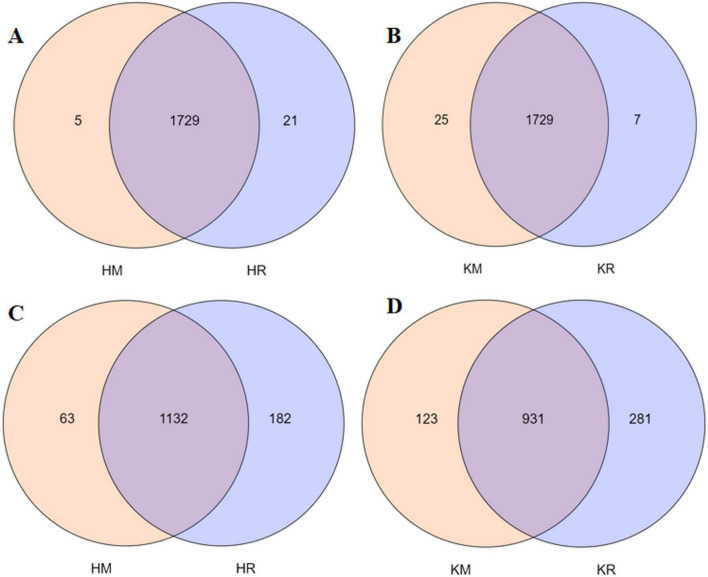
Venn diagram of bacterial and fungal communities in the tobacco monoculture and tobacco-maize relay intercropping pattern. Capital **(A,B)** represent bacterial communities of Hongda and K326, **(C,D)** represent fungal communities of Hongda and K326, respectively.

Venn diagrams intuitively display the common and unique OTUs between samples, illustrating their overlap. At 97% similarity, the OTU count per treatment was determined (Figure 4). In total, 1,736, 1,750, 1,754, and 1,736 bacterial OTUs and 1,195, 1,314, 1,054, and 1,212 fungal OTUs were obtained from Hongda monoculture, Hongda intercropped with maize, K326 monoculture, and K326 intercropped with maize, respectively. Across treatments, 1,729 bacterial OTUs were shared, with 5, 21, 25, and 7 unique to Hongda monoculture, Hongda intercropped with maize, K326 monoculture, and K326 intercropped with maize, respectively (Figures 4A, B). For fungal OTUs, 1,132 were shared between treatments and 63, 182, 123, and 281 were unique to the aforementioned treatments, respectively (Figures 4C, D). The results showed that rhizosphere soil microorganisms in the tobacco–maize relay intercropping patterns model shared similarities, yet exhibited larger differences than single-crop soil samples.

### 3.5 Soil microbial community richness and diversity under tobacco–maize relay intercropping

**FIGURE 5:**
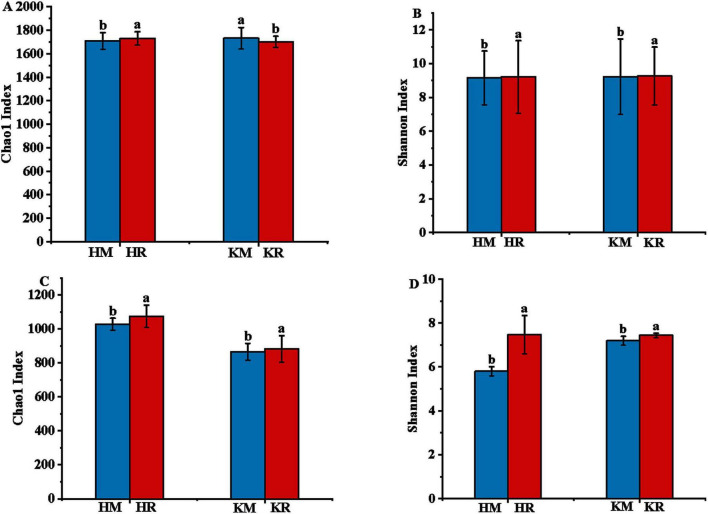
Sequence similarity of 97% for alpha diversity operational taxonomic units (OTUs) of bacterial communities in the tobacco monoculture and tobacco-maize relay intercropping pattern based on Illumina MiSeq sequencing. Different letters indicate significant differences (*P* < 0.05). Capital **(A,B)** represent bacterial community richness and diversity indices of tobacco, **(C,D)** represent fungal community richness and diversity indices of tobacco, respectively.

The diversity and richness of soil microbial communities are often characterized using the microbial diversity index and richness index, revealing differences in species and functions. Alpha diversity analysis of soil microbial communities employs the Chao1 index to reflect community species abundance and the Shannon index to reflect species diversity. Figure 5 illustrates that the Chao1 index of the soil bacterial community in Hongda intercropped with maize was significantly higher (*P* < 0.05) than in Hongda monoculture, with a 1.36% increase. In contrast, the Chao1 index of the maize soil bacterial community in K326 intercropping decreased by 1.62% compared with K326 monoculture, with no significant difference in the Shannon index (*P* > 0.05). Notably, the Chao1 index and Shannon index of the soil fungal community in Hongda intercropped with maize were significantly higher than in Hongda monoculture, increasing by 5.31 and 28.05%, respectively (*P* < 0.05). Similarly, the soil fungal community Chao1 index and Shannon index of K326 intercropped with maize were significantly higher (*P* < 0.05) than in K326 monoculture, increasing by 3.36 and 2.79%, respectively. These findings suggest that tobacco–maize relay intercropping enhances the richness and diversity of microbial communities in both cultivars.

### 3.6 Beta diversity of the soil microbial community under tobacco–maize relay intercropping

**FIGURE 6:**
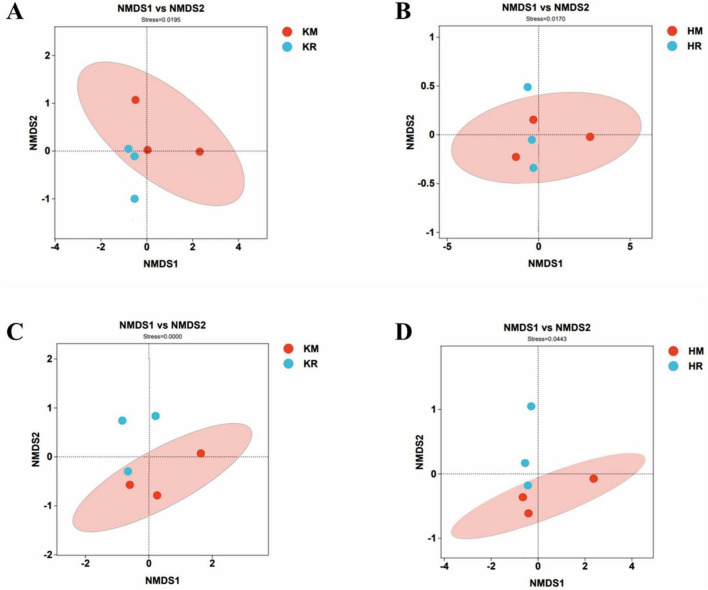
Differences in NMDS model bacterial and fungal community structure of soil microorganisms in the tobacco monoculture and tobacco-maize relay intercropping pattern. Differences in bacterial community structure between Saffron Daikin Won and K326 were **(A,B)**. Differences in the structure of Hongda and K326 fungal communities were **(C,D)**.

Figure 6 showed the beta diversity of soil microorganisms under tobacco–maize relay intercropping. An acceptable reliability in the non-metric multidimensional scaling (NMDS) analysis is indicated by a stress value of < 0.2. Figure 6 displays the NMDS results at the OTU level, with the stress values for both bacterial and fungal communities being 0.000. The above analysis showed that the soil microbial community structure under the tobacco–maize relay intercropping pattern revealed similarities to the single-crop flue-cured tobacco soil microbial community structure.

### 3.7 Taxonomic composition of soil bacterial and fungal communities under tobacco–maize relay intercropping

**FIGURE 7:**
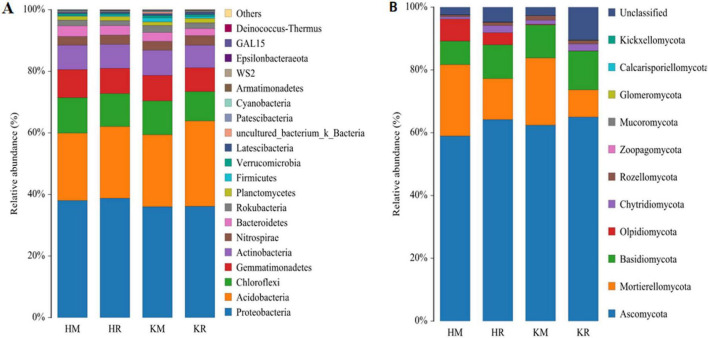
Species composition of soil bacteria and fungi at Phylum level under different planting patterns. Capital **(A,B)** represent bacterial community and fungal community at Phylum level, respectively.

In this study, we analyzed the composition of soil microorganisms at the phylum level. The inter-rhizosphere soil bacterial taxa of Hongda were primarily concentrated in the phyla Proteobacteria (38.06–38.77%), Acidobacteria (21.88–23.31%), Chloroflexi (10.71–11.51%), Gemmatimonadetes (8.19–9.12%), Actinobacteria (7.77–7.93%), Nitrospirae (2.84–3.03%), and Bacteroidetes (3.07–3.45%). The K326 inter-rhizosphere soil bacterial taxa were primarily concentrated in the phyla Proteobacteria (36.02–36.15%), Acidobacteria (23.39–27.73%), Chloroflexi (9.55–10.99%), Gemmatimonadetes (7.74–8.28%), Actinobacteria (7.30–8.16%), Nitrospirae (2.92–3.15%), and Bacteroidetes (2.28–2.88%). Among them, Proteobacteria, Bacteroidetes, Actinobacteria, Acidobacteria, Chloroflexi, and Nitrospirae contained the dominant species in the inter-rhizosphere soil of roasted tobacco in each treatment. Among the dominant bacterial groups, while Proteobacteria, Acidobacteria, and Nitrospirae species showed significantly higher relative abundances (*P* < 0.05), those of Actinobacteria, Chloroflexi, and Gemmatimonadetes species were significantly lower (Figure 7A) when compared with that of monoculture roasted tobacco. Nitrospirae and Acidobacteria were beneficial microorganisms in all treatments.

At the fungal phylum level (Figure 7B), the rhizosphere soil of Hongda was dominated by Ascomycota (58.96–64.19%), Mortierellomycota (13.06–22.74%), Basidiomycota (7.49–10.78%), Olpidiomycota (3.87–7.03%), Chytridiomycota (0.74–2.26%), and Glomeromycota (0.02–0.03%). Similarly, the inter-rhizosphere soil of K326 predominantly comprised Ascomycota (62.41–65.01%), Mortierellomycota (8.66–21.40%), Basidiomycota (10.56–12.36%), Olpidiomycota (0.09–0.2%), Chytridiomycota (1.31–2.20%), and Glomeromycota (0.03–0.04%). Notably, Peridiomycetes and Cladosporium mycorrhizal fungi, among these phyla, were beneficial microorganisms. Among the dominant bacterial species, the relative abundances of Ascomycota, Basidiomycota, and Chytridiomycota were significantly higher under the tobacco–maize relay intercropping patterns compared to the tobacco monoculture (Figure 7B).

Tobacco–maize relay intercropping significantly enhanced the overall abundance of beneficial microorganisms in the soil bacterial community and certain soil fungal communities for both cultivars. Furthermore, this intercropping method also increased soil microbial community diversity and stability, particularly for beneficial microbial populations such as arbuscular mycorrhizal fungi (AMF).

### 3.8 Redundancy analysis (RDA) of soil nutrients and soil microbial communities under tobacco–maize relay intercropping

**FIGURE 8:**
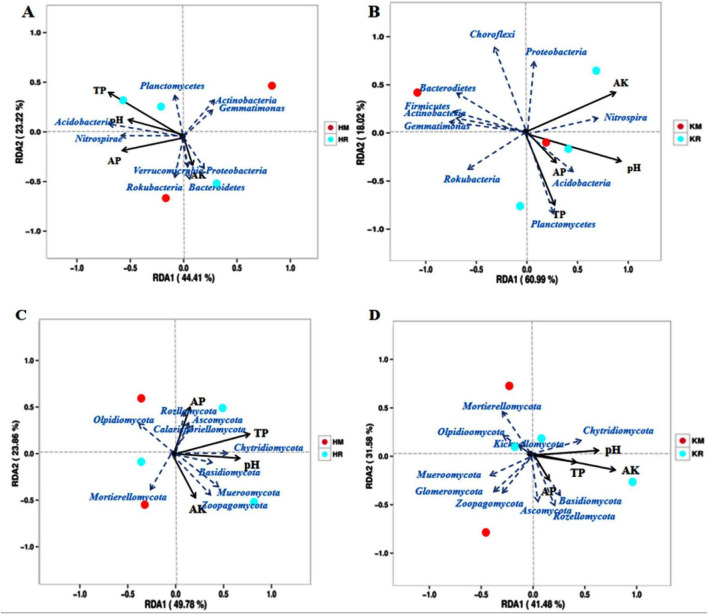
Redundancy analysis of roasted tobacco inter-root soil chemical properties with bacterial and fungal communities (Phylum level). Relationships between Hongda and K326 bacterial communities and environmental variables were **(A,B)**. Relationships between Hongda and K326 fungal communities and environmental variables **(C,D)**. Solid lines represent soil chemical properties and dashed lines represent bacterial species groups.

To elucidate the impact of soil’s physicochemical factors on microbial communities, a RDA was conducted on soil bacteria and fungi at the phylum level (Figure 8). The RDA revealed that the first two axes of the K326 bacterial community accounted for 56.79% of the community variation, with the first and second axes explaining 31.01 and 25.78%, respectively (Figure 8A). Similarly, the first two axes of the Hongda bacterial community RDA accounted for 64.20% of the community variation, with the first and second axes explaining 36.58 and 27.62%, respectively (Figure 8B). Notably, soil available potassium and nitrogen exerted a greater influence on the shifts in the soil bacterial community.

The fungal community structure’s RDA revealed that the K326 fungal community’s first two axes accounted for 61.17% of the community variation, with the first and second axes explaining 33.15 and 28.02%, respectively (Figure 8C). Similarly, the first two axes of the Hongda fungal community’s RDA accounted for 64.20% of the community variation, with the first and second axes explaining 36.58 and 27.62%, respectively (Figure 8D). Soil available potassium and nitrogen exerted a greater influence on soil bacterial community changes, with soil pH following. In summary, soil available phosphorus strongly influenced the soil microbial community structure. Both bacterial and fungal community structures exhibited significant positive correlations with total phosphorus, available phosphorus, and available potassium. Soil pH was positively and negatively correlated with bacterial and with the fungal community structures, respectively.

To understand the relationship between environmental factors and soil microbial communities, a correlation analysis was conducted between dominant bacterial groups at the phylum level and soil physicochemical properties (Table 2). The results indicated that all dominant bacterial groups, except the Proteobacteria, Nitrospirae, and Ascomycota, were significantly correlated with some physicochemical indexes. Specifically, Acidobacteria showed a highly significant positive correlation with available phosphorus (*P* < 0.01) and available potassium (*P* < 0.05). Actinobacteria exhibited a highly significant negative correlation with available phosphorus (*P* < 0.01). Basidiomycota had a significant positive correlation with available phosphorus (*P* < 0.05), while Chloroflexi, Gemmatimonadetes, and Mortierellomycota had a significant negative correlation with available phosphorus (*P* < 0.05). Glomeromycota was the only group that showed a highly significant positive correlation with total phosphorus (*P* < 0.01).

**TABLE 2:** Correlation between soil dominant flora (Phylum level) and physicochemical properties of flue-cured tobacco rhizosphere soil.

Phylum	AP	AK	TP	TK	pH
Proteobacteria	−0.347	−0.588	−0.111	−0.401	0.203
Acidobacteria	0.841**	0.670*	0.059	0.209	0.251
Chloroflexi	−0.712*	−0.603	−0.041	−0.118	−0.375
Gemmatimonadetes	−0.695*	−0.579	−0.019	−0.143	−0.493
Actinobacteria	0.810**	−0.445	−0.079	0.045	−0.343
Nitrospirae	0.583	0.416	0.118	−0.185	0.224
Ascomycota	0.565	0.309	−0.095	0.043	0.116
Mortierellomycota	−0.749*	−0.306	0.225	0.052	−0.267
Basidiomycota	0.639*	0.415	−0.129	0.600	0.075
Glomeromycota	−0.062	0.604	0.807**	0.139	0.160

* was significantly correlated at 0.05 level, and ** was significantly correlated at 0.01 level.

## 4 Discussion

Relay intercropping is a crucial planting pattern utilized globally to enhance crop yield (Wu et al., 2022). Most studies in this field have demonstrated that relay intercropping can improve soil resource utilization efficiency and boost farmers’ income (Tan et al., 2021; Li et al., 2019). In relay intercropping systems, the primary issue affecting root nutrient absorption, soil nutrient utilization, and crop growth is competition or sharing of soil nutrient resources (Tan et al., 2021; Liu et al., 2021). Our study found that the tobacco–maize relay intercropping pattern improved soil nutrients, specifically soil available phosphorus, available potassium, and available nitrogen, while decreasing soil total nitrogen and total potassium compared with tobacco monoculture. These results suggest that the tobacco–maize relay intercropping pattern offers a slight improvement in soil nutrient supply compared to tobacco monoculture. The plants’ absorption of total nitrogen, total potassium, and total phosphorus was enhanced. Intercropping patterns, as suggested previously, can improve the soil microenvironment, thereby promoting crops’ absorption and utilization of soil nutrients (Nwaogu et al., 2010). In this study, tobacco–maize relay intercropping intensified competition for nitrogen and potassium, while enhancing the soil nutrient supply of phosphorus. This demand rule aligned with the absorption of nitrogen and phosphorus by tobacco plants, indicating that nitrogen supply should be reduced and phosphorus supply increased during the tobacco harvesting stage. This adjustment ensures tobacco quality, offers a key approach to improve soil nutrients, and provides a theoretical basis for soil nutrient management in tobacco fields.

Soil enzyme activity significantly influences soil nutrients. This study demonstrated that tobacco–maize intercropping enhanced soil enzyme activities, notably urease and sucrase activities, while reducing catalase activity compared to tobacco monoculture. Fu et al. (2017) corroborated that inter-species interactions substantially enhance soil enzyme structure and activity. Thus, relay intercropping can improve soil enzyme activities, facilitating crop nutrient absorption. This may be attributed to the influence of soil enzyme activities on microbial communities (Ditterich et al., 2016). Prevalent research suggests that soil microbial communities impact soil enzyme activities, thereby enhancing the functional diversity of these communities and underscoring their crucial role in shaping soil enzyme activities and physicochemical properties (Dahal and Kim, 2016; Dari, 2019). Thus, in conclusion, soil microbial communities play a pivotal role in soil ecosystems, regulating nutrient cycling and serving as indicators of soil function (Yang et al., 2021). Most studies demonstrated that the size, activity, and structure of these communities changed with nitrogen fertilization (Li et al., 2020; Wang et al., 2023; Yevdokimov et al., 2008). The present study found that tobacco–maize relay intercropping enhances soil microbial composition, richness, and diversity. Specifically, the richness and diversity of the fungal community under tobacco–maize relay intercropping were significantly greater than those under tobacco monoculture, while bacterial community diversity remained largely unaffected.

The richness and diversity of soil microbial communities can enhance soil functions in ecosystems (Cameron et al., 2014). Previous studies have shown that relay intercropping can improve soil microbial community diversity, structure, and composition, thereby positively affecting crop growth and development (Qin et al., 2017; Xu et al., 2013). In the present study, the root system of maize secreted exudates that improved the soil microenvironment under tobacco-maize relay intercropping. Consequently, the soil microbial community composition was altered, and the metabolic activity of soil microorganisms was enhanced, thereby facilitating the diversification of soil microbial community structures (Rahman et al., 2021). Furthermore, soil microflora play a crucial role in soil carbon and nitrogen cycling and organic matter decomposition, thus helping to adapt to the new soil microenvironment and improve subsoil fertility. In this study, the tobacco-maize relay intercropping pattern had a soil bacterial community composition dominated by Proteobacteria, Acidobacteria, Bacillus, Chloroflexi, Nitrospira, and Bacteroidetes. Among them, the tobacco–maize relay intercropping pattern exhibited a higher abundance of Acidobacteria compared to tobacco monoculture. Acidobacteria plays an important role in soil nutrient cycling. Therefore, this suggests that this intercropping pattern can enhance soil nutrients, thereby influencing crop growth and development. The soil fungi community under this pattern primarily comprised Ascomycota, Mortierellomycota, Basidiomycota, and Chytridiomycota, with Ascomycota showing higher abundance than in tobacco monoculture. As a saprophytic fungus, Ascomycota significantly contributes to soil nutrient cycling as a decomposer. Notably, the genus *Sordaria* within Ascomycota, can decompose lignin in the subsoil by activating fungal laccase (Yang et al., 2020). Collectively, these observations suggest that relay intercropping benefits soil microorganisms by enriching community diversity and composition, and subsequently enhances soil nutrients through cellular laccase activation.

Environmental factors in soil significantly influence microbial community diversity (Degrune et al., 2019). This study’s RDA analysis revealed that soil microbial community composition was significantly affected by soil pH, available phosphorus, and available potassium. The soil bacterial and fungal community structures were primarily shaped by available phosphorus, aligning with previous research (Xu et al., 2020; Ng et al., 2014; Williams et al., 2006). Zhou et al. (2015) suggested that soil pH directly or indirectly impacts the structure and diversity of soil bacterial communities, thereby altering community composition. Our findings also indicated a positive correlation between soil pH and bacterial community structure, while the fungal community structure showed a negative correlation with soil pH. These results underscore the strong influence of pH on the structure of soil microbial communities and soil physicochemical properties. The study demonstrated that available phosphorus, available potassium, and total phosphorus were associated with dominant bacterial groups (Jisha and Mathur, 2005). Notably, Acidobacteria exhibited a positive correlation with available phosphorus content. This aligns with Saleemi’s research, which indicates that Acidobacteria are linked to phosphorus levels and significantly aid plants in phosphorus absorption. Conversely, Chloroflexi and Mortierellomycota were significantly negatively correlated with available phosphorus. This could potentially be attributed to changes in the composition and quantity of root exudates, leading to an increase in beneficial soil microbes and enhanced nutrient absorption in crops when tobacco was relay-intercropped with maize (Qi et al., 2009; Li et al., 2020; Rios-Velasco et al., 2015).

The study demonstrated that intercropping modified soil microbial community composition and soil physicochemical properties. However, the complexity of soil quality necessitates further investigation through soil microbiota transplant experiments and isolation of altered microbial taxa to elucidate the relationship between soil microbial community and soil quality.

## 5 Conclusion

This study’s findings indicate that the tobacco–maize relay intercropping pattern influences soil nutrients. Compared to tobacco monoculture, this intercropping pattern decreases total nitrogen and total potassium, yet increased the total phosphorus, available nitrogen, available phosphorus, and available potassium. RDA reveals a close association between soil physicochemical properties and microbial community structure. The richness and diversity of the soil microbial community, as indicated by the Chao1 and Shannon indices, respectively, are higher in the tobacco–maize relay intercropping pattern than in tobacco monoculture. Therefore, soil physicochemical properties, specifically soil pH, available phosphorus, and available potassium, are closely linked to dominant soil bacterial and fungal community structures. Then, the tobacco–maize relay intercropping pattern also enhances soil nutrient utilization and soil enzyme activities. These results suggest that the tobacco–maize relay intercropping pattern enhances soil microbial diversity, improves physicochemical properties, and boosts soil enzyme activity in the soil microenvironment, thereby increasing plant uptake of soil nutrients. Therefore, this provides a theoretical foundation for managing soil nutrients in tobacco fields.

## Data Availability

The data presented in the study are deposited in the BMKcloud repository with accession number: CRA021414.
